# MRI of Focal Liver Lesions

**DOI:** 10.2174/157340512800672216

**Published:** 2012-05

**Authors:** Nils Albiin

**Affiliations:** Division of Radiology, Karolinska Institutet, Karolinska University Hospital, Stockholm, Sweden

**Keywords:** Diagnosis, Diffusion weighted Magnetic Resonance Imaging, Hepatocellular carcinoma, HCC, Liver metastases, Liver neoplasms, Magnetic Resonance Imaging, MRI, Radiology, Review.

## Abstract

Magnetic resonance imaging, MRI has more advantages than ultrasound, computed tomography, CT, positron emission tomography, PET, or any other imaging modality in diagnosing focal hepatic masses. With a combination of basic T1 and T2 weighted sequences, diffusion weighted imaging, DWI, and hepatobiliary gadolinium contrast agents, that is gadobenate dimeglumine (Gd-BOPTA) and gadoxetic acid (Gd-EOB), most liver lesions can be adequately diagnosed. Benign lesions, as cyst, hemangioma, focal nodular hyperplasia, FNH or adenoma, can be distinguished from malignant lesions. In a non-cirrhotic liver, the most common malignant lesions are metastases which may be hypovascular or hypervascular. In the cirrhotic liver hepatocellular carcinoma, HCC, is of considerable importance. Besides, intrahepatic cholangiocarcinoma and other less common malignancies has to be assessed. In this review, the techniques and typical MRI features are presented as well as the new algorithm issued by American Association for the Study of the Liver Diseases (AASLD).

In patients with liver tumors, it is crucial to detect and stage the tumors at an early stage (to select patients who will benefit from curative liver resection, and avoid unnecessary surgery). Therefore, an optimal preoperative evaluation of the liver is necessary, and a contrast-enhanced MRI is widely considered the state-of-the-art method [1]. Liver MRI without contrast administration is appropriate for cholelithiasis but not sufficient for most liver tumor diagnoses.

In this review, a technical overview is followed by a presentation of the most common focal liver lesions.

## METHODS

### Non Contrast-Enhanced MRI

#### T1 and T2 Weighted Sequences

**Fig. (1):**
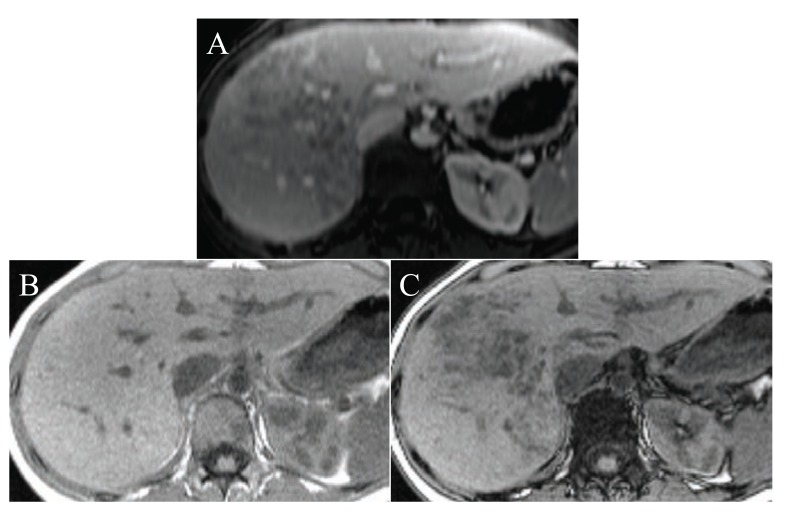
In the liver right lobe of a 48 years old woman, there are multiple hypointense lesions on T1 in the portovenous phase, after gadolinium injection (**A**). On T1 in phase the lesions are not visible (**B**), whereas on T1 out of phase the lesions are hypointense (**C**). This is typical for focal fatty infiltration or steatosis.

The non enhanced T1 and T2 weighted sequences are decisive in the characterization of focal liver diseases in cirrhotic as well as non cirrhotic liver. High signal intensity, SI, on T1 can be caused by bleeding, fat or deposition of copper or glycogen and can be seen in dysplastic nodules and hepatocellular carcinoma, HCC [2]. Most benign tumors are bright on T2w imaging, whereas malignant are slightly hyperintense (see below). On out–of–phase (opposed-phase) T1 imaging, abnormal fat accumulation in the liver will be hypointense, and is more accurate than ultrasound and CT to diagnose steatosis, focal fatty infiltration (Fig. **1**) and focal fatty sparing of the liver. It is also valuable in diagnosis of iron storage diseases - the iron containing liver parenchyma is hypointense on in-phase and isointense on out-of-phase [3].

#### Diffusion Weighted Imaging, DWI

**Fig. (2):**
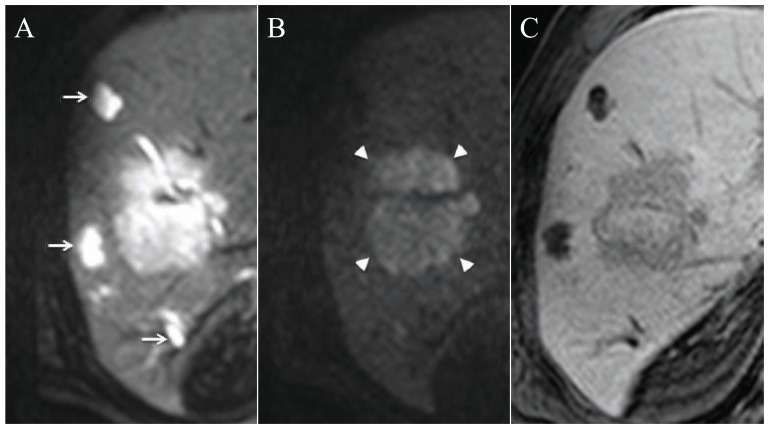
A patient with a large liver metastasis (from colorectal cancer) and three cysts. On DWI (A, B) the cysts (arrows) have a benign pattern with a hyperintensity on b=50 (A) and an isointensity on b=500 (B) compared to the metastasis (arrow heads) which has a malignant pattern with a hyperintensity also on b=500. In the hepatobiliary phase (2 hours after injection of Gd-BOPTA) the cysts are more hypointense compared to the metastasis.

Diffusion weighted echo-planar imaging, DWI, has been shown to be a reliable method to detect liver metastases (Fig. **2**), with a sensitivity and specificity better than multidetector CT, 87% and 97% for DWI compared with 53% and 78% for CT [4]. In a review by Bruegel and Rummeny [1] it was concluded that DWI is more sensitive than T2-weighted MRI and at least as accurate as superparamagnetic iron oxide (SPIO) or gadolinium-enhanced MR imaging for the detection of hepatic metastases [1]. It has also been claimed that apparent diffusion coefficient (ADC) measurement has the potential to discriminate benign and malignant focal hepatic lesions [5]. However, this has been questioned [6, 7]. A problem is that the reproducibility of ADC measurements is limited when using different imaging techniques (choice of different b-values and other acquisition parameters) and differences in scanner technology [1]. Among other limitations, it has to be pointed out that cystic or necrotic metastases, which show relatively high ADC values, may be false negative. Atypical hemangiomas with uncommonly low ADC values may be false positive. In addition, small lesions are difficult to distinguish due to partial volume effects and suboptimal region of interest (ROI) measurements, which may adulterate the ADC values [1]. In a larger study by Miller *et al.*, it was shown that cysts have significantly higher values compared to hemangiomas which has significantly higher ADC than solid lesions - however, the solid lesions could not be distinguished from one another, using the ADC values [7]. 

To conclude, DWI is an excellent diagnostic tool for the detection of even sub-centimeter liver lesions, and when further characterization is needed, a complementary contrast-enhanced imaging study should be performed. This is probably the most efficient preoperative evaluation [1]. DWI is also of value to identify lymph nodes and other extrahepatic lesions [7]. 

### Contrast-Enhanced MRI

#### Non Liver Specific, Gadolinium Enhanced MRI

There are several gadolinium, Gd, based extracellular contrast agents, without liver specific enhancement, and they have been extensively used for dynamic MRI (including native, late arterial, portal venous and equilibrium phases). Solid and vascularized lesions enhance whereas cystic and necrotic does not. ”Hypervascularized” lesions enhances intensely and early, in contrast to ”hypovascularized” tumors enhancing less and later (see below). 

### Liver Specific Contrast Enhancement

#### Hepatobiliary MRI

##### Hepatobiliary Gadolinium Enhanced MRI

Hepatobiliary contrast agents can be used to detect metastases (Fig. **2**), characterize liver lesions (with or without hepatocyte function/uptake) and to evaluate biliary excretion. There are two different hepatobiliary paramagnetic gadolinium chelates, gadobenate dimeglumine (Gd-BOPTA; MultiHance^®^, Bracco Imaging, Milan, Italy) and gadoxetic acid (Gd-EOB; Primovist^®^, Bayer Schering Pharma AG, Berlin, Germany). Gd-BOPTA has higher relaxivity values and SI changes than the non liver specific Gd-based contrast agents but similar safety profile [8, 9]. Gd-EOB has a rapid and abundant biliary excretion (50%, in comparison to 5% for Gd-BOPTA) [10], and is, therefore, readily used for functional studies [11, 12]. In contrast, the vascular enhancement with Gd-EOB is less pronounced and has a short duration [13-15]. The low dynamic enhancement of Gd-EOB may be increased with a slower injection rate [16]. The hepatobiliary phase is usually sufficient at 20 min after Gd-EOB and 1-2 hours after Gd-BOPTA administration. Thus, the two hepatobiliary contrast agents have different profiles. At our department, we use Gd-BOPTA when the dynamic enhancement in different phases is more important than the hepatobiliary function, and vice versa, we use Gd-EOB when the hepatobiliary function is more crucial than the dynamic Gd-MRI.

T2-weighted magnetic resonance cholangiopancreatography (MRCP) sequences should not be obtained after administration of Gd-EOB-DTPA, because the rapid biliary excretion of this contrast agent decreases SI of the biliary structures on T2 [17]. However, some obtain MRCP both before and after Gd-EOB, and if the high SI decreases or if the high SI disappears, it is interpreted as evidence that bile secretion works. DWI may be performed after Gd-EOB without compromising contrast to noise ratio and ADC of focal hepatic lesions [18].

##### Future: Response After Treatment

Dynamic contrast-enhanced MRI, DCE-MRI, has been analyzed to determine the effect of drugs on tumor angiogenesis and vascular disruption. Although early results show that this is beneficial, its practical application is far from straightforward. A review of the literature [20] has pointed out that these acquisition and analyses are immensely complex. Furthermore, it was concluded that, before DCE-MRI can serve as a new surrogate endpoint, further research and validation to clinical outcome are needed.

Experience in diffusion-weighted MRI in monitoring treatment response has shown promising results [21, 22]. Recommendations on standardization of the application have recently been published [23] and further studies are needed.

### Benign Tumors

The most common benign tumors have typical imaging features. Knowledge of these findings and the clinical history are essential in correctly diagnosing focal liver lesions. However, it is important to know that common liver tumors may manifest with atypical findings, posing a diagnostic dilemma when they do [24]. 

#### Cysts

Congenital biliary cysts are common with incidence up to 14%. A typical hepatic cyst is unilocular with a nearly imperceptible wall and a homogeneous content with a remarkably low SI on T1 and extremely high SI on T2 weighted images (white as liquor in the spine and bile in the gallbladder) which increases on heavily T2 weighted images. Cysts do not enhance after contrast injection [25]. The diagnostic accuracy for MRI is 97% compared to 67% for CT [26] and is even more confident using DWI [7] (Fig. **2**). 

#### Hemangioma

**Fig. (3):**
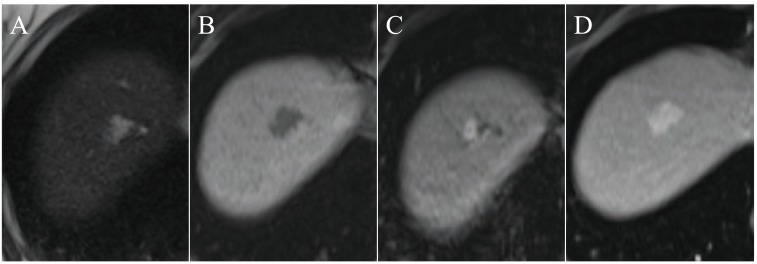
In the upper dome of the right liver lobe there is a typical hemangioma with a high signal on T2 (**A**) and low signal on T1 (**B**). After gadolinium contrast injection, there is nodular enhancement in the arterioportal phase (**C**) and filling in, in late phase (D; 5 minutes after contrast injection).

Cavernous hemangioma is the most common hepatic tumor, incidence 20%. A typical hemangioma is well delineated and hypointense as blood on T1w images and clearly hyperintense on T2w images (Fig. **3**). The contrast enhancement is peripheral and nodular in early phases, followed by progressive centripetal filling in late and delayed phases. The SI is similar to blood. Small hemangiomas, <2 cm, may have homogeneous enhancement in late arterial phase and resemble hepatocellular carcinoma and hypervascular metastasis. However, hemangiomas follow SI of blood [25]. Hemangiomas has an ADC significantly higher than solid liver lesions and lower than cysts [7].

#### Focal Nodular Hyperplasia, FNH

Focal nodular hyperplasia, FNH, is a response to a vascular anomaly with incidence up to 8%. FNH is asymptomatic and an incidental finding in up to 90%, more commonly seen in young to middle aged women and is solitary in 80%. Oral contraceptives have been suggested to stimulate growth, however, in a study the size and the number of FNH lesions were not influenced by oral contraceptive use [27].

**Fig. (4):**
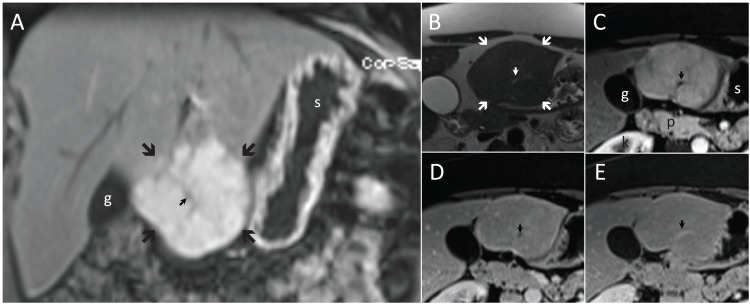
Typical focal nodular hyperplasia, FNH (large arrows), in coronal (**A**) and transverse planes (**B**-**E**). It is slightly hyperintense to the liver on T2 (**B**) and enhances richly on T1 in the arterial phase (**A** and **C**) followed by isointensity in the delayed phases (**D** and **E**). Note the central scar (small arrow), which is hyperintense on T2 (**B**) and hypointense on T1 in arterial (**A** and **C**) and portal venous (**D**) phases, whereas hyperintense after 5 minutes (**E**). g, gallbladder; k, right kidney; p, pancreas; s, stomach.

**Fig. (5):**
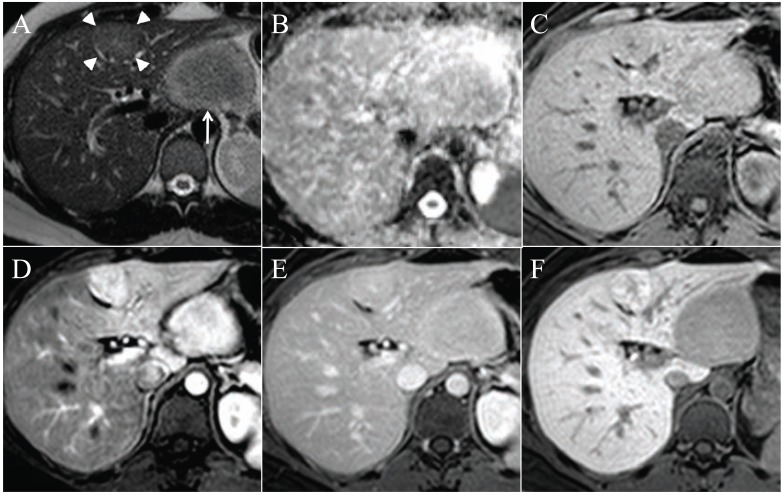
In the left liver lobe there is both a focal nodular hyperplasia (arrow heads) and an adenoma (arrow). Both lesions are iso- or slightly hyperintense on T2 (**A**), iso- or slightly hypointense on ADC-mapping (**B**), iso- or slightly hypointense on T1 gradient echo before gadolinium contrast (**C**), hyperintense in the arterioportal phase (**D**) and isointense in the venous/delayed phase. Note in the hepatobiliary phase (**F**; MultiHance, 2 hours post injection), the FNH is hyperintense, whereas the adenoma is hypointense to the liver parenchyma.

A typical FNH is hyperintense in the arterial phase, and isointense before contrast and in the venous phase. Thin radiating septa divide the tumor, but there is no capsule. A majority of FNH (89%) is before contrast hyper- to isointense on T2 and iso- to hypointense on T1. After gadolinium administration, 98% of FNH show a rapid and intense enhancement during the arterial phase, followed by a hyper- to isointensity in portal venous and equilibrium phases (Fig. **4**). Accurate discrimination of FNH from hepatic adenoma is not possible on this imaging alone. However, if using hepatobiliary contrast enhancement an accurate differentiation is achievable on delayed T1, 1-3 hours after gadobenate dimeglumine enhancement: FNH appear hyper-isointense (97%), whereas adenomas are hypointense (100%) [28] (Fig. **5**). 

Half of FNH has a central scar, slightly hyperintense on T2 and with late Gd-contrast enhancement (Fig. **4**, in contrast to fibrolamellar HCC with hypointense scar without contrast enhancement). A critical note is that, on Gd-EOB enhanced imaging, the central scar of focal nodular hyperplasia lesions does not typically demonstrate delayed enhancement [29]. Still, FNH is better characterized with Gd-EOB than with CT [30].

On DWI, the ADC values of FNH and adenomas are similar to metastases and HCC limiting the value for differentiating solid liver masses [7].

Atypical FNH - lacking central scar and being hyperintense on both T2w and T1w images (both natively and in delayed phases) and isointense in the hepatobiliary phase - need complementary studies, biopsy and/or follow up for correct diagnosis. 

#### Hepatocellular Adenoma, HCA

Hepatocellular adenoma, HCA, is in the West a rare tumor of women on oral contraceptive (85-98%) or men on anabolic steroids. Risk of hemorrhage increases in adenomas larger than 4-5 cm and during pregnancy. However, evidence of minimal intratumoral bleeding is often present. Some adenomas are steatotic, and may be multifocal (60%) and even coexisting with FNH. HCAs are considered premalignant but rarely develops into HCC unless larger than 10 cm (10% risk). Those with male hormone administration, familial polyposis and mutated b–catenin has enhanced risk [33].

A typical adenoma has heterogeneous SI on both T1 and T2 weighted sequences due to hemorrhage, necrosis and/or steatosis. The enhancement is after Gd-contrast heterogeneous in the arterial phase and hypointense in the hepatobiliary phase [28] (Fig. **5**). 

DWI can’t be used reliably to differentiate adenoma from FNH or HCC [6, 7].

#### Nodules in Cirrhotic Liver

It is considered that regenerative nodules, RN, can stepwise develop into hepatocellular carcinoma, HCC, via low-grade dysplastic nodules (DN), high-grade DN and well-differentiated HCC. In this development, from benign to malignant, the portal venous supply diminishes and the arterial supply increases. Regenerative nodules are hypointense on T1 and T2 weighted images. Dysplastic nodules (and ⅓ of HCC) are hyperintense on T1 weighted images [32]. 

A focal lesion that is hyperintense on T1w/T2w imaging is a potential HCC and must be further characterized [33]. In a cirrhotic liver, small lesions (≤2 cm) with arterial enhancing on T1 without hyperintensity on T2 were found to be benign in a study on explanted liver [34]. Arterial enhancement, venous wash out and/or decreased hepatocyte function speak in favor of HCC (see chapter below). On DWI, 79% of DN are iso- or hypointense whereas 97% of HCCs are hyperintense [35].

Lobar or segmental atrophy and confluent fibrosis are hypointense on T1 and hyperintense on T2 weighted imaging. After Gd, the enhancement is delayed and may finally be hyperintense to the liver parenchyma.

### Malignant Tumors

#### Hepatocellular Carcinoma, HCC

##### Background

Hepatocellular carcinoma (HCC) is a common primary tumor in the world and the third most common cause of cancer related death, after lung and stomach cancer. Identification of HCC at an early stage is crucial for prompt surgical resection or transplantation [36]. Therefore, risk groups with cirrhosis (chronic viral hepatitis B/C, alcoholism and/or non-alcoholic steatohepatitis) are in surveillance programs, and the recommendation is still surveillance with ultrasound. However, the chance to find small HCC is dismal. In a recent surveillance study, the sensitivity of contrast-enhanced ultrasound, CT and MRI for small HCC, 1-2cm, was 26%, 44% and 44% respectively [37]. The recommendations and algorithms for detected lesions have changed during the last decade [38-40]. According to the practice guidelines issued by the American Association for the Study of the Liver Diseases (AASLD) in 2010, the diagnosis of HCC must rest on radiology and histology. Thus, a mass in a cirrhotic liver with an elevation of AFP is no longer automatically indicating HCC as it can also be due to cholangiocarcinoma or metastasis from colorectal cancer [40]. According to the homepage of the European Association for the Study of the Liver (EASL) they will also meet with new recommendations.

##### Standard Diagnosis

**Fig. (6):**
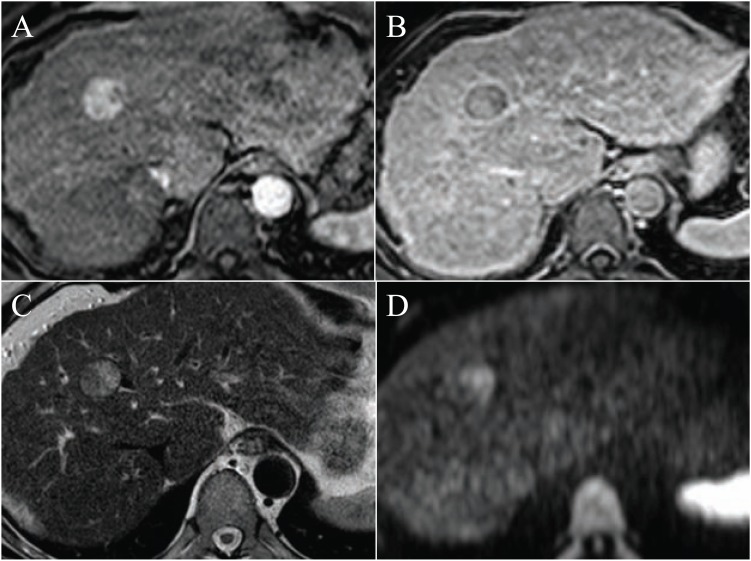
A 55 years old man with hepatitis B has a hypervascular lesion in the right lobe of the cirrhotic liver. On T1, in the arterial phase (**A**), it is hyperintense, followed by hypointense washout in portovenous phase (**B**). On T2 it is slightly hyperintense (**C**) and on DWI, b=500, it is also hyperintense (**D**) – characteristic findings for hepatocellular carcinoma, HCC.

The standard of care for the radiological diagnosis of HCC in cirrhosis is dynamic contrast imaging exhibiting typical arterial enhancement and venous/late washout [41] (Fig. **6**). According to the new AASLD recommendations, for lesions ≥1 cm, characteristic findings with arterial hypervascularity and venous washout, in one proper examination, including a dynamic multidetector CT or an MRI, with 4 phases (unenhanced, arterial, venous and delayed), is diagnostic for HCC without biopsy. This apply to lesions in cirrhotic liver and to patients with chronic hepatitis B but not to nodules in otherwise normal liver. However, it is essential to point out that HCC cannot be ruled out by the absence of arterial hypervascularization or absence of delayed washout. The false-negative tumors should be diagnosed on follow-up imaging before the lesion reaches a size where the chance of cure is diminished [40].

The accuracy of imaging diagnosis of HCC in liver cirrhosis depends largely on the degree of arterial hypervascularization, which increases with tumor size and grade of malignancy [42]. Large HCC, >2 cm, show typical hypervascularity in the arterial phase and washout in the portal venous and/or equilibrium phase. The lesion based sensitivity of MRI, in patients with cirrhosis who received liver transplants, was 72% overall sizes, compared to 65% with CT and 46% with ultrasound [43]. For small tumors, 1-2 cm, the sensitivity for MRI is low, only 44–47%, which is equivalent to CT (40–44%) but higher than contrast-enhanced ultrasound, 21–26% [37, 43]. Low grade HCC, 1-2 cm, showed typical enhancing features in only two of 16 whereas high grade in 17 of 31 HCC [37]. There are more recent studies indicating that MRI with gadolinium has the highest sensitivity and specificity for small HCC [44-49].

The correlation of HCC size and sensitivity was readily shown in a Gd BOPTA study correlated to explanted livers and HCC. Overall there was an excellent sensitivity and specificity of 87% and 79% respectively and a positive predictive value of 65-66% in the detection of HCC. However, the size was crucial for the sensitivity: >2 cm 100%, 1-2 cm 83-89%, ≥1 cm 91-94%, <1 cm 29-43%. False positive lesions measuring 1-2 cm were dysplastic nodules (85-100%), and <1 cm were nonneoplastic arterial hypervascular lesions (80-86%). The delayed phase imaging had limited diagnostic value due to poor hepatocyte function and enhancement in this group of late cirrhosis [50].

Large HCC are heterogeneous and regularly invades the portal vein, and this is a significant prognostic factor (regulating recurrence and survival in HCC patients, treated with resection or orthotopic liver transplantation) [55].

##### Alternate Approaches

In a cirrhotic liver, a solid lesion hyperintense on T2 is suspected for HCC [32]. A hypointense lesion on dynamic Gd-MRI on T1 but slightly hyperintense on T2, has been regarded as HCC in some studies [52, 53]. However, HCC is a chameleon and can in a cirrhotic liver mimic hemangioma, adenoma, FNH and hypervascular metastases, and can even be isointense in the arterial phase and lack wash out in venous phases. 

A main challenge now is to better characterize hypervascular nodules <2 cm, which often have nonspecific imaging characteristics [54]. One option to help differ HCC from arterial-enhancing pseudolesion may be hepatobiliary MRI. In a recent study, in the hepatobiliary phase, 95% of HCC were hypointense on T1, whereas 94% of the pseudolesions were isointense. The sensitivity was 91–94% compared with 54% with CT [55]. A solid lesion lacking hepatobiliary uptake has been claimed to be an additional criterion for HCC, especially in contrast to regenerative nodules [56]. In another study on HCC ≤2 cm, it was shown that by adding DWI to conventional dynamic Gd-MRI, the sensitivity was significantly increased from 85% to 98% [57].

On DWI, a typical HCC is hyperintense on images with high b-values and is dark (restricted diffusion) on ADC image. By adding DWI to CE-MRI the accuracy to differentiate HCC from dysplastic nodule increases from 75% to 93% [35]. 

These alternate approaches may improve the diagnosis of small lesions. However, prospective studies, with accurate imaging-pathology correlations on explanted livers, are warranted before any alternate criterion is approved as the standard diagnostic technique for HCC. 

#### Fibrolamellar Hepatocellular Carcinoma

Fibrolamellar HCC is rare and seen without underlying cirrhosis. A typical fibrolamellar HCC is lobulated, large (usually >12 cm) with a calcified central scar, and has metastases in ⅔ of cases. It differs from FNH in several aspects: enhances heterogeneously and the scar is hypointense on T2w and does not enhance in arterial and venous phases and only partially on delayed imaging [62]. Conventional HCC in non cirrhotic liver can mimic fibrolamellar HCC but lacks central scar.

#### Cholangiocarcinoma

**Fig. (7):**
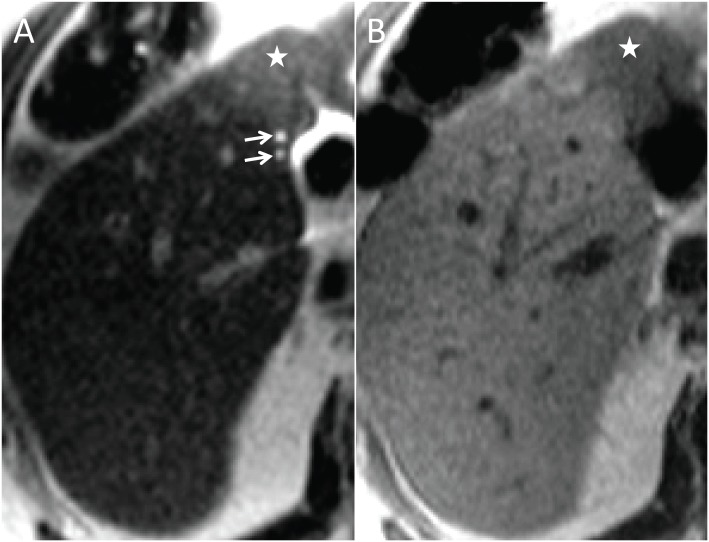
In the left liver lobe there is a cholangiocarcinoma, hyperintense on T2 (**A**) and hypointense on T1 (**B**). Note dilated bile ducts in the periphery of the tumor (arrows).

Intrahepatic cholangiocarcinoma is quite rare and is less common than hilar/Klatskin tumors (90%). It is associated with repeated/chronic cholangitis (*e.g.* primary sclerosing cholangitis) and cystic disease of the liver. A typical intrahepatic cholangiocarcinoma is hyperintense on T2w images and often obstruct vessels and bile ducts, with upstream ductal dilatation (Fig. **7**). In affected segment(s), there is a volume loss with capsular retraction. After contrast, the enhancement is delayed, starting in the periphery, resembling hemangioma. However, the enhancement of cholangiocarcinoma is not isointense to vessels. In addition, intrahepatic cholangiocarcinomas may also display homogeneous arterial contrast enhancement [59] - interestingly, these tumors, examined using contrast-enhanced ultrasound, there was a late wash out, mimicking hepatocellular carcinoma.

Early diagnosis is a challenge and there is hope through new technique: in a study on patients with biliary stricture, proton magnetic resonance spectroscopy, 1H-MRS, of bile, it was possible to discriminate cholangiocarcinoma from benign biliary conditions [60]. If this will come true *in vivo*, early diagnosis might be possible. 

#### Biliary Cystadenocarcinoma

Benign biliary cystadenoma rarely transforms into cystadenocarcinoma. A typical cystadenocarcinoma is multiloculated with enhancing nodules and septa, and calcifications.

#### Metastases

Metastases are the most common malignant liver tumors and may be hypo- or hypervascular, best diagnosed with gadolinium dynamically. However, in cirrhotic livers metastases are rare [61].

##### Hypovascular Metastases

Most hepatic metastases are multiple, hypointense on T1 and hyperintense on T2 and hypovascular in dynamic contrast imaging. Cystic metastases may be intensely bright on T2 and resemble cysts, abscesses and hemangiomas. There is contrast enhancement in the periphery but the center is hypointense. After hepatobiliary contrast metastases are hypointense on T1 in a bright liver.

Dynamic Gd-contrast-enhanced MRI is more sensitive than contrast-enhanced CT, as sensitive as FDG-PET in depicting liver metastases from colorectal cancer, and more specific in lesion characterization [62].

Hepatobiliary contrast agents may even further increase the sensitivity of MRI for diagnosis of colorectal cancer liver metastases [15]. This may be particularly useful after neoadjuvant chemotherapy when metastases become difficult to diagnose due to changed tumor vascularity and liver steatosis [15]. Gd-EOB has higher accuracy to detect small liver metastases than DWI. However, by combining the Gd-EOB and DWI the detection may be improved [63].

DWI is more sensitive than T2-weighted MRI for the detection of hepatic metastases. Hepatic metastases show higher SI on DWI than on T2-weighted fast SE images, whereas the signal from vessels and cysts are suppressed with DWI. Small lesions occasionally need further characterization [1, 64] (Fig. **2**).

##### Hypervascular Metastases

**Fig. (8):**
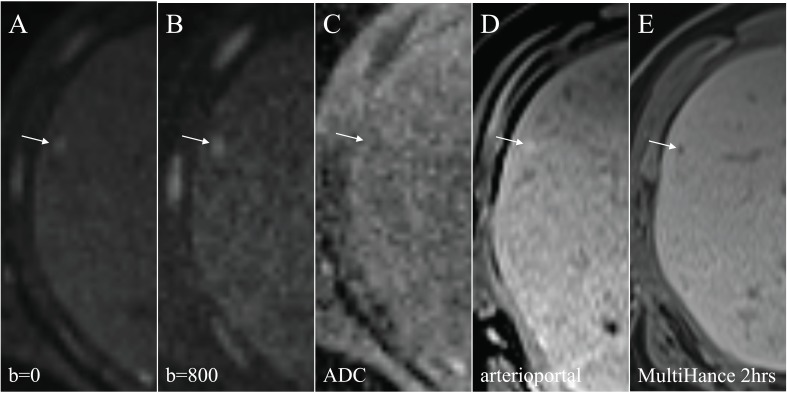
A carcinoid liver metastasis (arrows) is hyperintense and readily detected on DWI, and the intensity is less pronounced on b=0 (**A**) compared to b=800 (**B**). On ADC mapping, the lesion is dark with a low ADC value and is hard to detect and delineate, probably due to the small size of the lesion (**C**). After Gd contrast injection, on T1 in the arterioportal phase, the tumor is hyperintense due to its hypervascularity (**D**). In the hepatobiliary phase, on T1, it is clearly hypointense (**E**).

Liver metastases from renal cell carcinoma, neuroendocrine tumors, sarcoma, malignant melanoma, thyroid carcinoma, some breast and colorectal cancer are hyperintense in late arterial phase imaging [65] (Fig. **8**). They are often multiple and do not follow the SI of vessels. On T2, the SI of hypervascular metastases is usually moderately elevated, and on T1 hypointense. Melanoma is an exception, where melanin accumulation may result in hyperintensity on T1. There are also common benign hypervascular liver lesions, hemangioma, focal nodular hyperplasia and some tumor-like liver conditions. On DWI, metastases with a marked hypervascularity can attain high ADC values [7] and be false negative.

#### Lymphoma

Secondary liver lymphoma is multiple, and primary lymphoma, which is rare, is solitary [66]. Lymphoma is isointense to the spleen and hypointense on T1 and hyperintense on T2 compared to surrounding liver tissue. After contrast enhancement lymphoma is hypointense centrally with a peripheral enhancement. In the hepatobiliary phase, the lesions are hypointense and may have a target-like appearance [67].

#### Non-Neoplastic Lesions

##### Hydatid

In echinococcosis, there is a large cystic mass with numerous peripheral daughter cysts. Calcifications, if present, are hard to see on MRI. Cyst wall and septa enhances after Gd-contrast injection. On DWI, hydatid cysts with restricted diffusion and low ADC value, differ from simple cysts, with free diffusion and high ADC value [5].

##### Liver Abscess

**Fig. (9):**
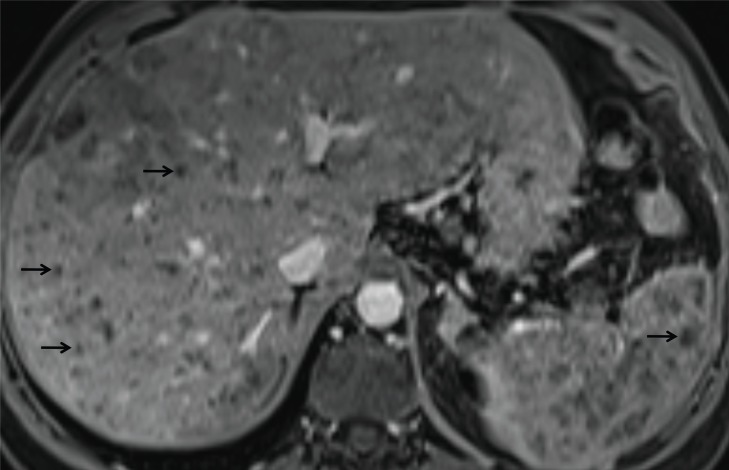
Candidiasis with multiple microabscesses (a few are indicated with arrows) in both the liver and the spleen. The abscesses are in the portal-venous phase hypointense on T1.

Most liver abscesses are pyogenic and can be portal or biliary in origin (Fig. **9**). Typically, cluster of small abscesses coalesce into a large cavity with air or fluid level and is surrounded by an enhancing capsule. Small abscesses (<1 cm) may enhance contrast and mimic hemangiomas. Metastases, necrotic after treatment, may be indistinguishable from abscesses. However, DWI studies have indicated that DWI could discriminate abscess and cystic or necrotic tumors (abscess has lower ADC values when compared with the necrotic portions of tumors) [5, 68].

## LIST OF ABBREVIATIONS

AASLD: = American Association for the Study of the Liver Diseases
ADC: = apparent diffusion coefficient
CT: = computed tomography
DCE-MRI: = dynamic contrast-enhanced MRI
DN: = dysplastic nodules
DWI: = diffusion weighted imaging
EASL: = European Association for the Study of the Liver
FNH: = focal nodular hyperplasia
Gd: = gadolinium
Gd-BOPTA: = gadobenate dimeglumine
Gd-EOB: = gadoxetic acid
HCA: = hepatocellular adenoma
HCC: = hepatocellular carcinoma
MRCP: = magnetic resonance cholangiopancreatography
MRI: = magnetic resonance imaging
MRS: = magnetic resonance spectroscopy
PET: = positron emission tomography
ROI: = region of interest
SI: = signal intensity

## References

[1] Bruegel M, Rummeny EJ (2010). Hepatic metastases: use of diffusion-weighted echo-planar imaging. Abdom Imaging.

[2] Matsui O, Kadoya M, Kameyama T (1989). Adenomatous hyperplastic nodules in the cirrhotic liver: differentiation from hepatocellular carcinoma with MR imaging. Radiology.

[3] Merkle EM, Nelson RC (2006). Dual gradient-echo in-phase and opposed-phase hepatic MR imaging: a useful tool for evaluating more than fatty infiltration or fatty sparing. Radiographics.

[4] Holzapfel K, Reiser-Erkan C, Fingerle AA (2010). Comparison of diffusion-weighted MR imaging and multidetector-row CT in the detection of liver metastases in patients operated for pancreatic cancer. Abdom Imaging.

[5] Kilickesmez O, Bayramoglu S, Inci E, Cimilli T (2009). Value of apparent diffusion coefficient measurement for discrimination of focal benign and malignant hepatic masses. J Med Imaging Radiat Oncol.

[6] Sandrasegaran K, Akisik FM, Lin C, Tahir B, Rajan J, Aisen AM (2009). The value of diffusion-weighted imaging in characterizing focal liver masses. Acad Radiol.

[7] Miller FH, Hammond N, Siddiqi AJ (2010). Utility of diffusion-weighted MRI in distinguishing benign and malignant hepatic lesions. J Magn Reson Imaging.

[8] Shellock FG, Parker JR, Pirovano G (2006). Safety characteristics of gadobenate dimeglumine: clinical experience from intra- and interindividual comparison studies with gadopentetate dimeglumine. J Magn Reson Imaging.

[9] Schneider G, Maas R, Schultze Kool L (2003). Low-dose gadobenate dimeglumine versus standard dose gadopentetate dimeglumine for contrast-enhanced magnetic resonance imaging of the liver: an intra-individual crossover comparison. Invest Radiol.

[10] Reimer P, Schneider G, Schima W (2004). Hepatobiliary contrast agents for contrast-enhanced MRI of the liver: properties, clinical development and applications. Eur Radiol.

[11] Dahlstrom N, Persson A, Albiin N, Smedby O, Brismar TB (2007). Contrast-enhanced magnetic resonance cholangiography with Gd-BOPTA and Gd-EOB-DTPA in healthy subjects. Acta Radiol.

[12] Nilsson H, Nordell A, Vargas R, Douglas L, Jonas E, Blomqvist L (2009). Assessment of hepatic extraction fraction and input relative blood flow using dynamic hepatocyte-specific contrast-enhanced MRI. J Magn Reson Imaging.

[13] Brismar TB, Dahlstrom N, Edsborg N, Persson A, Smedby O, Albiin N (2009). Liver vessel enhancement by Gd-BOPTA and Gd-EOB-DTPA: a comparison in healthy volunteers. Acta Radiol.

[14] Tamada T, Ito K, Sone T (2009). Dynamic contrast-enhanced magnetic resonance imaging of abdominal solid organ and major vessel: comparison of enhancement effect between Gd-EOB-DTPA and Gd-DTPA. J Magn Reson Imaging.

[15] Seale MK, Catalano OA, Saini S, Hahn PF, Sahani DV (2009). Hepatobiliary-specific MR contrast agents: role in imaging the liver and biliary tree. Radiographics.

[16] Zech CJ, Vos B, Nordell A (2009). Vascular enhancement in early dynamic liver MR imaging in an animal model: comparison of two injection regimen and two different doses Gd-EOB-DTPA (gadoxetic acid) with standard Gd-DTPA. Invest Radiol.

[17] Nakamura Y, Ohmoto T, Saito T, Kajima T, Nishimaru E, Ito K (2009). Effects of gadolinium-ethoxybenzyl-diethylenetriamine pentaacetic acid on T2-weighted MRCP. Magn Reson Med Sci.

[18] Choi JS, Kim MJ, Choi JY, Park MS, Lim JS, Kim KW (2010). Diffusion-weighted MR imaging of liver on 3.0-Tesla system: effect of intravenous
administration of gadoxetic acid disodium. Eur Radiol.

[19] Semelka RC, Helmberger TK (2001). Contrast agents for MR imaging of the liver. Radiology.

[20] O'Connor JP, Jackson A, Parker GJ, Jayson GC (2007). DCE-MRI biomarkers in the clinical evaluation of antiangiogenic and vascular disrupting agents. Br J Cancer.

[21] Yu JS, Kim JH, Chung JJ, Kim KW (2009). Added value of diffusion-weighted imaging in the MRI assessment of perilesional tumor recurrence after chemoembolization of hepatocellular carcinomas. J Magn Reson Imaging.

[22] Yuan Z, Ye XD, Dong S (2010). Role of magnetic resonance diffusion-weighted imaging in evaluating response after chemoembolization of hepatocellular carcinoma. Eur J Radiol.

[23] van Persijn van Meerten EL, Gelderblom H, Bloem JL (2009). RECIST revised: implications for the radiologist A review article on the modified RECIST guideline. Eur Radiol.

[24] Anderson SW, Kruskal JB, Kane RA (2009). Benign hepatic tumors and iatrogenic pseudotumors. Radiographics.

[25] Federle MP G-CS, Anne VS, MP F (2005). Hepatobiliary and Pancreas. Diagnostic Imaging Abdomen.

[26] Sasaki K, Ito K, Fujita T (2007). Small hepatic lesions found on single-phase helical CT in patients with malignancy: diagnostic capability of breath-hold, multisection fluid-attenuated inversion-recovery (FLAIR) MR imaging using a half-fourier acquisition single-shot turbo spin-echo (HASTE) sequence. J Magn Reson Imaging.

[27] Mathieu D, Kobeiter H, Maison P (2000). Oral contraceptive use and focal nodular hyperplasia of the liver. Gastroenterology.

[28] Grazioli L, Morana G, Kirchin MA, Schneider G (2005). Accurate differentiation of focal nodular hyperplasia from hepatic adenoma at gadobenate dimeglumine-enhanced MR imaging: prospective study. Radiology.

[29] Karam AR, Shankar S, Surapaneni P, Kim YH, Hussain S (2010). Focal nodular hyperplasia: central scar enhancement pattern using Gadoxetate Disodium. J Magn Reson Imaging.

[30] Zech CJ, Grazioli L, Breuer J, Reiser MF, Schoenberg SO (2008). Diagnostic performance and description of morphological features of focal nodular hyperplasia in Gd-EOB-DTPA-enhanced liver magnetic resonance imaging: results of a multicenter trial. Invest Radiol.

[31] Bioulac-Sage P, Laumonier H, Laurent C, Zucman-Rossi J, Balabaud C (2008). Hepatocellular adenoma: what is new in 2008. Hepatol Int.

[32] Kadoya M, Matsui O, Takashima T, Nonomura A (1992). Hepatocellular carcinoma: correlation of MR imaging and histopathologic findings. Radiology.

[33] Shinmura R, Matsui O, Kobayashi S (2005). Cirrhotic nodules: association between MR imaging signal intensity and intranodular blood supply. Radiology.

[34] Holland AE, Hecht EM, Hahn WY (2005). Importance of small (< or = 20-mm) enhancing lesions seen only during the hepatic arterial phase at MR imaging of the cirrhotic liver: evaluation and comparison with whole explanted liver. Radiology.

[35] Xu PJ, Yan FH, Wang JH, Shan Y, Ji Y, Chen CZ (2010). Contribution of diffusion-weighted magnetic resonance imaging in the characterization of hepatocellular carcinomas and dysplastic nodules in cirrhotic liver. J Comput Assist Tomogr.

[36] Llovet JM (2005). Updated treatment approach to hepatocellular carcinoma. J Gastroenterol.

[37] Sangiovanni A, Manini MA, Iavarone M (2010). The diagnostic and economic impact of contrast imaging techniques in the diagnosis of small hepatocellular carcinoma in cirrhosis. Gut.

[38] Bruix J, Sherman M, Llovet JM (2001). Clinical management of hepatocellular carcinoma. Conclusions of the Barcelona-2000 EASL conference. European Association for the Study of the Liver. J Hepatol.

[39] Bruix J, Sherman M (2005). Management of hepatocellular carcinoma. Hepatology.

[40] Bruix J, Sherman M (2010). Management of Hepatocellular Carcinoma: An Update. AASLD PRACTICE GUIDELINE. Hepatology.

[41] Lencioni R, Crocetti L, Della Pina MC, Cioni D (2008). Guidelines for imaging focal lesions in liver cirrhosis. Expert Rev Gastroenterol Hepatol.

[42] Iavarone M, Sangiovanni A, Forzenigo LV (2010). Diagnosis of hepatocellular carcinoma in cirrhosis by dynamic contrast imaging: the importance of tumor cell differentiation. Hepatology.

[43] Yu NC, Chaudhari V, Raman SS (2010). Computed Tomography and Magnetic Resonance Imaging Improve Detection of Hepatocellular Carcinoma, Compared With Ultrasound Alone, in Patients With Cirrhosis. Clin Gastroenterol Hepatol.

[44] Golfieri R, Marini E, Bazzocchi A (2009). Small (<or=3 cm) hepatocellular carcinoma in cirrhosis: the role of double contrast agents in MR imaging vs. multidetector-row CT. Radiol Med.

[45] Kim SH, Choi BI, Lee JY (2008). Diagnostic accuracy of multi-/single-detector row CT and contrast-enhanced MRI in the detection of hepatocellular carcinomas meeting the milan criteria before liver transplantation. Intervirology.

[46] Lee MW, Kim YJ, Park HS (2010). Targeted sonography for small hepatocellular carcinoma discovered by CT or MRI: factors affecting sonographic detection. AJR Am J Roentgenol.

[47] Leoni S, Piscaglia F, Golfieri R (2010). The impact of vascular and nonvascular findings on the noninvasive diagnosis of small hepatocellular carcinoma based on the EASL and AASLD criteria. Am J Gastroenterol.

[48] Zech CJ, Reiser MF, Herrmann KA (2009). Imaging of hepatocellular carcinoma by computed tomography and magnetic resonance imaging: state of the art. Dig Dis.

[49] Kim SH, Lee J, Kim MJ (2009). Gadoxetic acid-enhanced MRI versus triple-phase MDCT for the preoperative detection of hepatocellular carcinoma. AJR Am J Roentgenol.

[50] Choi SH, Lee JM, Yu NC (2008). Hepatocellular carcinoma in liver transplantation candidates: detection with gadobenate dimeglumine-enhanced MRI. AJR Am J Roentgenol.

[51] Nishie A, Yoshimitsu K, Asayama Y (2008). Radiologic detectability of minute portal venous invasion in hepatocellular carcinoma. AJR Am J Roentgenol.

[52] Kelekis NL, Semelka RC, Worawattanakul S (1998). Hepatocellular carcinoma in North America: a multiinstitutional study of appearance on T1-weighted, T2-weighted, and serial gadolinium-enhanced gradient-echo images. AJR Am J Roentgenol.

[53] Hecht EM, Holland AE, Israel GM (2006). Hepatocellular carcinoma in the cirrhotic liver: gadolinium-enhanced 3D T1-weighted MR imaging as a stand-alone sequence for diagnosis. Radiology.

[54] Golfieri R, Marini E, Bazzocchi A (2009). Small (</=3 cm) hepatocellular
carcinoma in cirrhosis: the role of double contrast agents in
MR imaging vs. multidetector-row CT. Radiol Med.

[55] Sun HY, Lee JM, Shin CI (2010). Gadoxetic acid-enhanced magnetic resonance imaging for differentiating small hepatocellular carcinomas (< or =2 cm in diameter) from arterial enhancing pseudolesions: special emphasis on hepatobiliary phase imaging. Invest Radiol.

[56] Kim YK, Lee JM, Kim CS, Chung GH, Kim CY, Kim IH (2005). Detection of liver metastases: gadobenate dimeglumine-enhanced three-dimensional dynamic phases and one-hour delayed phase MR imaging versus superparamagnetic iron oxide-enhanced MR imaging. Eur Radiol.

[57] Xu PJ, Yan FH, Wang JH, Lin J, Ji Y (2009). Added value of breathhold diffusion-weighted MRI in detection of small hepatocellular carcinoma lesions compared with dynamic contrast-enhanced MRI alone using receiver operating characteristic curve analysis. J Magn Reson Imaging.

[58] Corrigan K, Semelka RC (1995). Dynamic contrast-enhanced MR imaging of fibrolamellar hepatocellular carcinoma. Abdom Imaging.

[59] Vilana R, Forner A, Bianchi L (2010). Intrahepatic peripheral cholangiocarcinoma in cirrhosis patients may display a vascular pattern similar to hepatocellular carcinoma on contrast-enhanced ultrasound. Hepatology.

[60] Albiin N, Smith IC, Arnelo U (2008). Detection of cholangiocarcinoma with magnetic resonance spectroscopy of bile in patients with and without primary sclerosing cholangitis. Acta Radiol.

[61] Uetsuji S, Yamamura M, Yamamichi K, Okuda Y, Takada H, Hioki K (1992). Absence of colorectal cancer metastasis to the cirrhotic liver. Am J Surg.

[62] Bipat S, van Leeuwen MS, Comans EF (2005). Colorectal liver metastases: CT, MR imaging, and PET for diagnosis--meta-analysis. Radiology.

[63] Shimada K, Isoda H, Hirokawa Y, Arizono S, Shibata T, Togashi K (2010). Comparison of gadolinium-EOB-DTPA-enhanced and diffusion-weighted liver MRI for detection of small hepatic metastases. Eur Radiol.

[64] Nasu K, Kuroki Y, Nawano S (2006). Hepatic metastases: diffusion-weighted sensitivity-encoding versus SPIO-enhanced MR imaging. Radiology.

[65] Namasivayam S, Salman K, Mittal PK, Martin D, Small WC (2007). Hypervascular hepatic focal lesions: spectrum of imaging features. Curr Probl Diagn Radiol.

[66] Gazelle GS, Lee MJ, Hahn PF, Goldberg MA, Rafaat N, Mueller PR (1994). US CT and MRI of primary and secondary liver lymphoma. J Comput Assist Tomogr.

[67] Beaty SD, Silva AC, Depetris G (2008). AJR teaching file: incidental hepatic mass. AJR Am J Roentgenol.

[68] Chan JH, Tsui EY, Luk SH (2001). Diffusion-weighted MR imaging of the liver: distinguishing hepatic abscess from cystic or necrotic tumor. Abdom Imaging.

